# Stilbene‐based derivatives as potential inhibitors of trimethylamine (TMA)‐lyase affect gut microbiota in coronary heart disease

**DOI:** 10.1002/fsn3.3046

**Published:** 2022-09-12

**Authors:** Jincai Li, Peng Huang, Wangxing Cheng, Qian Niu

**Affiliations:** ^1^ School of Traditional Chinese Medicine Bozhou University Bozhou China; ^2^ School of Pharmacy Anhui University of Chinese Medicine Hefei China; ^3^ Department of Pharmacy Bozhou Vocational and Technical College Bozhou China

**Keywords:** coronary heart disease, molecular docking, stilbenes, TMAO, trimethylamine‐lyase

## Abstract

Coronary heart disease (CHD) is defined by atherosclerosis, which may result in stenosis or blockage of the arterial cavity, leading to ischemic cardiac diseases such as angina and myocardial infarction (MI). Accumulating evidence indicates that the gut microbiota play a critical role in the initiation and progression of CHD. The gut microbial metabolite trimethylamine N‐oxide (TMAO) is intimately linked to the pathophysiology of CHD. The hepatic flavin‐containing monooxygenases (FMOs) convert trimethylamine (TMA) to TMAO. As a result, it is critical to prevent TMA generation. Stilbenes could reduce cardiovascular disease mortality. Twelve stilbenes with inhibitory activity against TMA‐lyase were compiled and evaluated in this study. Docking results showed Resveratroloside had the highest Vina score, indicating that it was the most active and might be employed as a lead molecule for further structural modification.

## Graphical abstract

Docking results showed Resveratroloside had the highest Vina score, indicating that it was the most active and might be employed as a lead molecule for further structural modification. The possible action process of TMA‐Lyase lead compound was described, which further affects the occurrence and development of CHD.

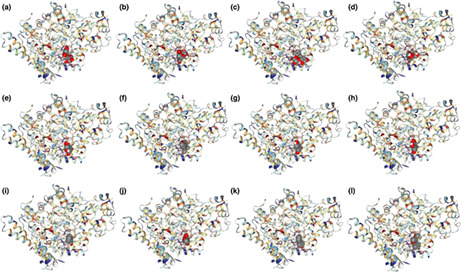

## 1 INTRODUCTION

Coronary heart disease (CHD) is a rising pandemic in developing countries and the top cause of mortality in many industrialized societies. Atherosclerosis is a risk factor for CHD, since it may result in stenosis or blockage of the vascular cavity, leading to ischemic heart diseases such as angina and myocardial infarction (MI) (Hu et al., 2020). Accumulating data indicate that the gut microbiota and its metabolites are crucial in the onset and development of CHD (Sun et al., 2019). Additionally, gut metagomic sequencing revealed an increase in the frequency of *Collinsella* bacteria in individuals with symptomatic atherosclerosis, while *Eubacterium* and *Roseburia* bacteria were more abundant in controls (Karlsson et al., 2012). The terminal restrictive fragment length polymorphism (T‐RFLP) and 16S ribosomal RNA (16S rRNA) were utilized to investigate changes in the gut microbiota of patients with CHD and healthy individuals. The findings indicated that individuals with CHD had a considerably increased number of mature lactobacilli, a significantly reduced number of *Bacteroides* (*Bifidobacterium* and *Prevotella*), and a significantly higher ratio of *Firmicutes*/*Bacteroides* (Emoto et al., 2017). The gut microbiota composition of 218 persons with CHD was shown to differ from that of healthy individuals due to an increase in the number of Enterobacteriaceae (*Escherichia coli*, *Klebsiella* spp.,*Enterobacter aerogenes*) and *Streptococcus* spp. (Jie et al., 2017). Human fecal 16S rRNA gene sequencing revealed a substantial reduction in the number of *Bacteroides vulgatus* and *Bacteroides dorei* in individuals with CHD. Atherosclerosis‐prone mice were intragastrically fed live *B. vulgatus* and*B. dorei*, which have been shown to reduce the formation of atherosclerotic lesions, alleviate endotoxemia, decrease the production of gut microbial lipopolysaccharide (LPS), and effectively inhibit pro‐inflammatory immune responses (Yoshida et al., 2018). *Bacteroides vulgatus* and *Bacteroides dorei* were shown to be beneficial in preventing atherosclerosis in the original fecal samples of patients with CHD (Yoshida et al., 2019). A multi‐omic investigation of 161 CHD patients and 40 healthy controls revealed that the makeup of the gut microbiota and metabolites varied considerably according to the severity of the CHD. The principal morphological variants includeRoseburia, *Klebsiella*, *Clostridium IV*, and Ruminococcaceae (Liu et al., 2019). As additional research demonstrates that gut microbiota is strongly associated with the incidence and progression of CHD, microbiota is predicted to become a critical target for CHD prevention and treatment (Liu et al., 2020a; Trøseid et al., 2020).

Accumulating data indicate that the gut microbiota and its metabolites play a critical role in the genesis and progression of CHD (Xu & Yang, 2020). The oral microbiome has the potential to alter the makeup of the gut microbiota and contribute to the development of various diseases. A specific diet and the use of prebiotics and probiotics have favorable effects on community structure optimization (Hills Jr et al., 2019). The fermentation metabolites of the gut microbiota, such as trimethylamine N‐oxide (TMAO), short‐chain fatty acids (SCFAs), and secondary bile acid (BA), have also been linked to the development, prevention, treatment, and prognosis of CHD (Verhaar et al., 2020). The gut microbiota's metabolic TMAO synthesis is a critical mechanism of cardiovascular disease. We will discuss the role of TMAO in CHD in this section.

Recent research has established a substantial correlation between plasma TMAO levels and CHD risk, with increasing TMAO levels associated with a considerable increase in the incidence of acute myocardial infarction (AMI), cardiogenic shock, and mortality (Otto & Rahimi, 2016). Clinical research discovered that patients with elevated plasma TMAO levels had a considerably higher 3‐year incidence of cardiovascular events, which may be due to the increased degree of atherosclerosis associated with TMAO (Tang et al., 2013). Reduced TMAO levels dramatically reduced the plaque area, increased the thickness of the fibrous cap, and decreased the amount of necrotic lipid cores in C57/BL6 mice unstable carotid plaque model, showing that reducing TMAO level can improve the stability of carotid atherosclerotic plaque (Shi et al., 2021). Additionally, TMAO can promote calcium ion entry into cells via adenosine diphosphate, thrombin, and collagen, increasing platelet sensitivity, which promotes thrombosis and may directly induce myocardial infarction (MI) (Li et al., 2021). These findings suggested that TMAO may be employed as a biomarker for CHD and hence as a viable research subject. Cholines in diet are digested by the choline utilization TMA‐lyase system [CutC/D] to generate TMA. TMA is then absorbed through the portal vein and processed further by the host's liver enzymes (FMOs, primarily flavin‐containing dimethylaniline monooxygenase 3 (FMO3)) to form TMAO (Gabr & Świderek, 2020; Heng et al., 2021). The TMA/TMAO pathway is one of the microbe‐dependent mechanisms that will eventually be connected to CHD pathogenesis (Skye et al., 2018). Recently, small molecule inhibitors of the primary bacterial TMA‐lyase enzymes have been designed on the basis of their mode of action. Thus, TMA plays a decisive role in the formation of TMAO. In other words, decreasing the quantity of TMAO by preventing the development of TMA‐producing bacteria may help alleviate CHD symptoms. Thus, inhibiting TMA‐lyase activity can result in a decrease in TMAO generation, and TMA‐lyase can be exploited as a target for further study.

Stilbene is a common structural scaffold found in nature, and molecules derived from stilbene have been extensively studied for their biological function. Notably, resveratrol and its naturally occurring stilbene‐containing derivatives have been widely studied as antioxidant, anti‐inflammatory, and anticancer agents (De Filippis et al., 2017; Feng et al., 2021; Pecyna et al., 2020). Numerous studies have revealed that stilbenes may help lower the mortality associated with cardiovascular diseases such as ischemic heart disease and heart failure (Frombaum et al., 2012). Stilbenes have an effect on the equilibrium of the gut microbiota, which is directly related to human health (Han et al., 2018). Because stilbenes protect the cardiovascular system and have the ability to regulate the gut microbiota, and the gut microbiota is directly related to TMAO, improved stilbene compounds were evaluated. Thus, lowering the quantity of TMAO involves preventing the transition of TMA to TMAO, which may be performed by inhibiting TMA synthesis. This requires inhibiting CutC choline TMA‐lyase activity. According to this study, it is critical to discover effective and safe medications that target TMA‐lyase in order to cure CHD. The purpose of this article is to screen stilbenes for safe, effective, and economically viable TMA‐lyase inhibitors.

## 2 STILBENES

### 2.1 Resveratrol

Resveratrol (RES) is a naturally polyphenolic phytoalexin discovered from *Veratrum grandiflorum* and abundant in grapes, wine, peanuts, soy, and berries, which has captivated scientists and physicians for decades (Breuss et al., 2019). Resveratrol revealed significant cardioprotective benefits in a rat model of AMI; probable mechanisms include enhanced cardiac function and atrial interstitial fibrosis through RES‐mediated regulation of the NLR family pyrin domain containing 3 (NLRP3) inflammasome and inhibition of the transforming growth factor beta 1 (TGF‐β1)/SMAD2 signaling pathway (Jiang et al., 2021). Resveratrol protected isolated rat heart or H9c2 cells against myocardial ischemia/reperfusion damage by upregulating vascular endothelial growth factor B (VEGF‐B), which also provided cardioprotection against isoproterenol (ISO)‐induced myocardial infarction (MI) via the VEGF‐B/AMPK/eNOS/NO (vascular endothelial growth factor B/AMP‐activated protein kinase/endothelial NO synthase/nitric oxide) signaling pathway (Feng et al., 2019). Resveratrol supplementation enhanced gut health by enhancing the function of the intestinal barrier, reducing intestinal inflammation and oxidative damage, and favorably modulating the gut microbiota (Qiu et al., 2021). In cardiovascular patients, resveratrol can increase intestinal epithelial integrity, modulate the intestinal microbiota, and decrease TMAO generation (Song et al., 2020).

### 2.2 Resveratroloside

Resveratroloside is a monoglucosylated stilbene found in red wine, grapes, and a variety of traditional medicinal herbs (Zhao et al., 2019). The results indicated that Resveratroloside may greatly improve the area of myocardial infarction (MI) following 30 min of ischemia and 120 min of reperfusion, elucidating Resveratroloside's cardioprotective function (Naumenko et al., 2013).

### 2.3 Rhaponticin

Rhaponticin is a hydroxystilbene found in the *rhubarb rhizomes*, which has been demonstrated to have a variety of biological actions, including anticancer, antihyperlipidemic, anti‐allergic, antioxidant, and antibacterial properties (Chen et al., 2020). Rhaponticin has been shown to preserve heart function by decreasing serum lactate dehydrogenase (LDH) and creatine kinase (CK) levels (Chen et al., 2009).

### 2.4 2,3,5,4′‐Tetrahydroxystilbene‐2‐O‐glucoside

2,3,5,4′‐Tetrahydroxystilbene‐2‐O‐glucoside is a resveratrol analog combined with glucoside that contains the active components recovered from *Polygonum multiflorum* Thunb. (Zhang & Chen, 2018). 2,3,5,4′‐Tetrahydroxystilbene‐2‐O‐glucoside exhibited potent anti‐inflammatory, anti‐oxidative, and anti‐apoptotic properties, resulting in prolonged effects on neuronal, cardiomyocyte, and endothelial cell damage (Wu et al., 2017). 2,3,5,4′‐Tetrahydroxystilbene‐2‐O‐glucoside enhanced the cardioprotective impact of temporary hypoxia on hypoxia/reoxygenation (H/R) by inhibiting excessive reactive oxygen species (ROS) generation and calcium overload via increased mitochondrial energy metabolism (Cheng et al., 2018), and significantly prevented the development of atherosclerotic (AS) plaques in apolipoprotein E (ApoE)−/− mice and decreased atherosclerosis (AS) through control of the total gut microbiota composition (Li et al., 2020).

### 2.5 Polydatin

Polydatin is a monocrystalline substance derived from the root and rhizome of *Polygonum cuspidatum*Sieb. et Zucc. that has antioxidant and anti‐inflammatory properties (Park et al., 2019). Furthermore, polydatin exhibited effects on hypertension, cardiac ischemia/reperfusion injury, hypertrophy, and heart failure (Liu et al., 2017). Polydatin was reported to dramatically enhance heart function in rats with AMI and to minimize hypoxia‐induced myocardial cell damage in in vivo and in vitro investigations, which was associated with promoting Nrf2/HO‐1 (nuclear factor erythroid 2‐related factor 2/heme oxygenase 1) signal transduction and decreasing oxidative stress injury (Chen et al., 2019). Polydatin also dramatically alleviated myocardial dysfunction and decreased histological abnormalities by increasing the left ventricular shortening fraction and ejection fraction and decreasing cardiac hypertrophy and interstitial fibrosis (Tan et al., 2020).

### 2.6 Piceatannol

Piceatannol is a naturally occurring polyphenol that is a hydroxylated analog of resveratrol. It exhibits a broad range of biological activity, including cancer prevention, cardioprotection, neuroprotection, antidiabetic, and depigmentation (Cao et al., 2020). Piceatannol restored the impaired cardiac function by inhibiting the JAK2/STAT3 (Janus kinase 2/signal transducer and activator of transcription 3) pathway in cecal ligation and puncture‐induced septicemia animals and LPS‐stimulated H9c2 cardiomyocytes (Xie et al., 2021). Piceatannol protects cardiomyocytes from peroxidative damage by reducing excessive ROS and calcium overload caused by H_2_O_2_, preventing mitochondrial depolarization, and upregulating PI3K–Akt–eNOS (phosphatidylinositol 3‐kinase/protein kinase B/endothelial NO synthase) signaling (Wang et al., 2019). Additionally, piceatannol can enhance the composition of the gut microbiota by increasing *Firmicutes* and *Lactobacillus* and lowering *Bacteroidetes*as compared to the high‐fat diet group (Tung et al., 2016).

### 2.7 Rhapontigenin

Rhapontigenin, a stilbene aglycone metabolite of rhaponticin isolated from the medicinal plant *rhubarb rhizomes*, has been found to possess a variety of biological activities, including anticancer, antihyperlipidemic, anti‐allergic, antioxidant, and anti‐inflammatory properties (An et al., 2021; Chen et al., 2020). Rhapontigenin has been shown to improve infarct size, heart/body weight index, CK, LD, and cardiac troponin‐T (CTT) in rats through its anti‐inflammatory, antioxidant, and anti‐apoptotic properties (Fan, 2019).

### 2.8 Pterostilbene

**FIGURE 1:**
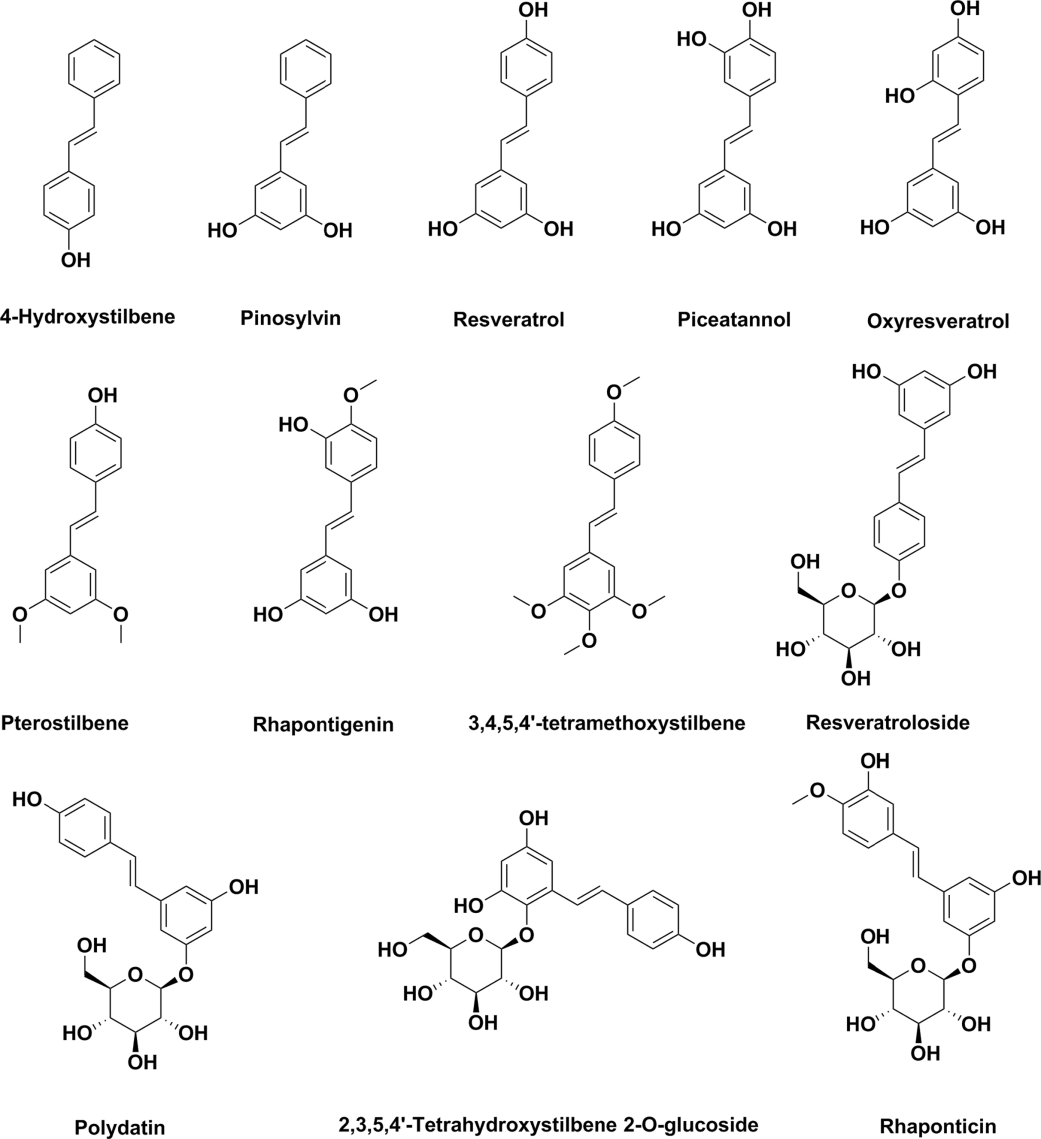
Chemical structures of stilbenes.

Pterostilbene is a resveratrol analog that has been reported to have similar and frequently more potent health‐promoting properties, implying that it may be used to treat diabetes, cardiovascular disease, fatty liver disease, and Alzheimer's disease (Kim et al., 2020; Lin et al., 2020). Pterostilbene significantly enhanced heart function and decreased myocardial infarction (MI) and apoptosis during myocardial ischemia/reperfusion damage (Yu et al., 2017). Pterostilbene modifies intestinal bacteria composition toward a healthier microbial profile, that is, increasing the abundance of Bacteroides and decreasing the plasma TMAO (Etxeberria et al., 2017; Koh et al., 2019) (Figure 1).

## 3 MOLECULAR DOCKING

CB‐Dock is a user‐friendly blind docking web server that can be used to anticipate a protein's binding sites and compute the center and size of the cavity using a curvature‐based cavity detection technique. Additionally, the procedure began with the identification of the target and the expected ligand chemical molecule. The procedure is as follows: (1) CutC choline trimethylamine lyase (TMA)‐lyase (Protein Data Bank (PDB) ID: 5A0U) (Kalnins et al., 2015) was obtained in PDB format from the Research Collaboratory for Structural Bioinformatics Protein Data Bank (RCSB PDB) database. (2) PubChem was used to acquire the chemical structures of stilbene aglycones and glycosides. (3) PDB formats of TMA‐lyase, stilbene aglycones, and glycosides were input to CB‐Dock for molecular docking (Liu et al., 2020b). The lower the Vina score, which means the stronger the binding ability between ligand and target.

**FIGURE 2:**
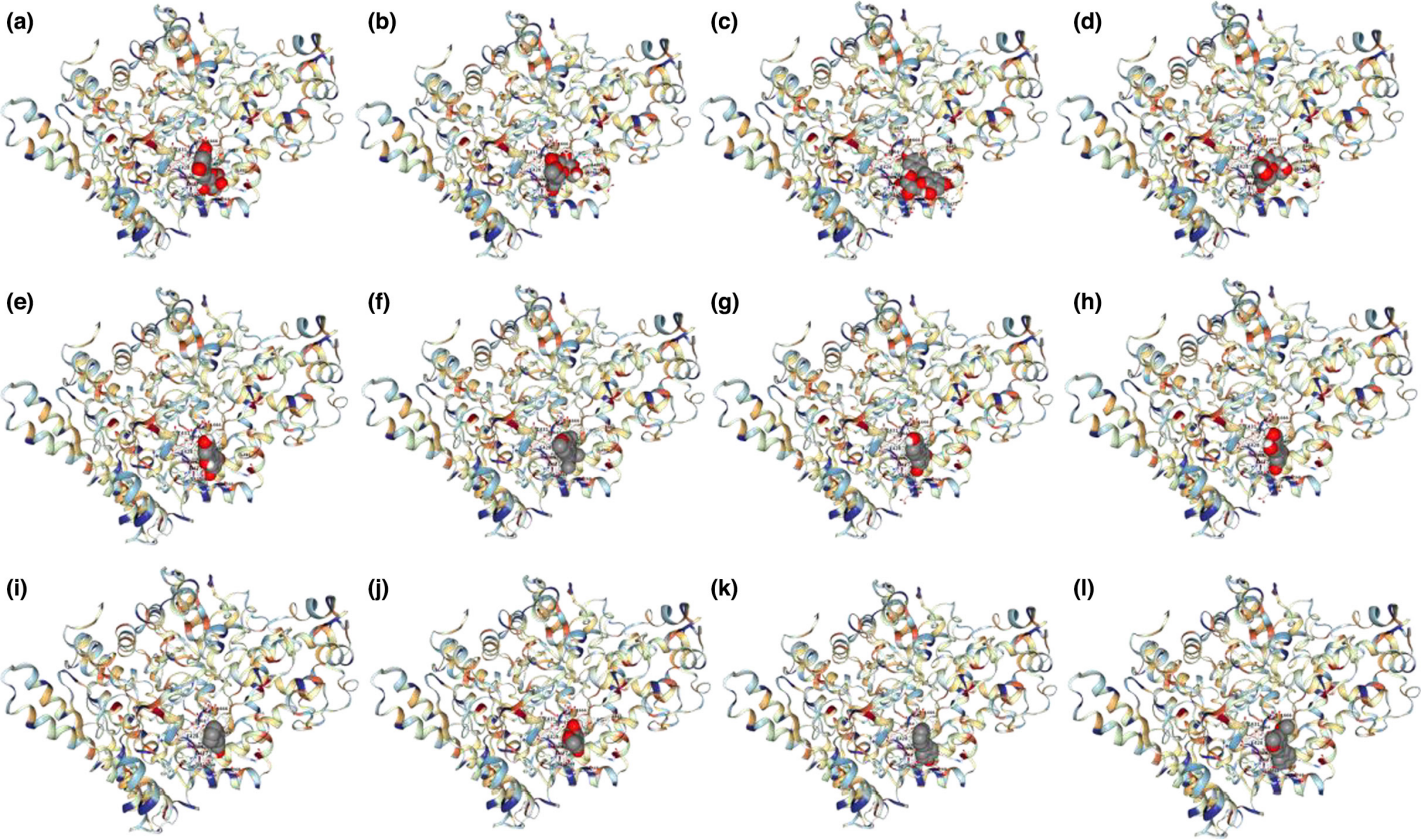
The three‐dimensional (3D) pictures of stilbene aglycones and glycosides with trimethylamine (TMA)‐lyase. (a) Resveratroloside, (b) Rhaponticin, (c) 2,3,5,4′‐Tetrahydroxystilbene 2‐O‐glucoside, (d) Polydatin, (e) Oxyresveratrol, (f) 3,4,5,4′‐Tetramethoxystilbene, (g) Resveratrol, (h) Piceatannol, (i) Pinosylvin, (j) Rhapontigenin, (k) 4‐Hydroxystilbene, (l) Pterostilbene.

**FIGURE 3:**
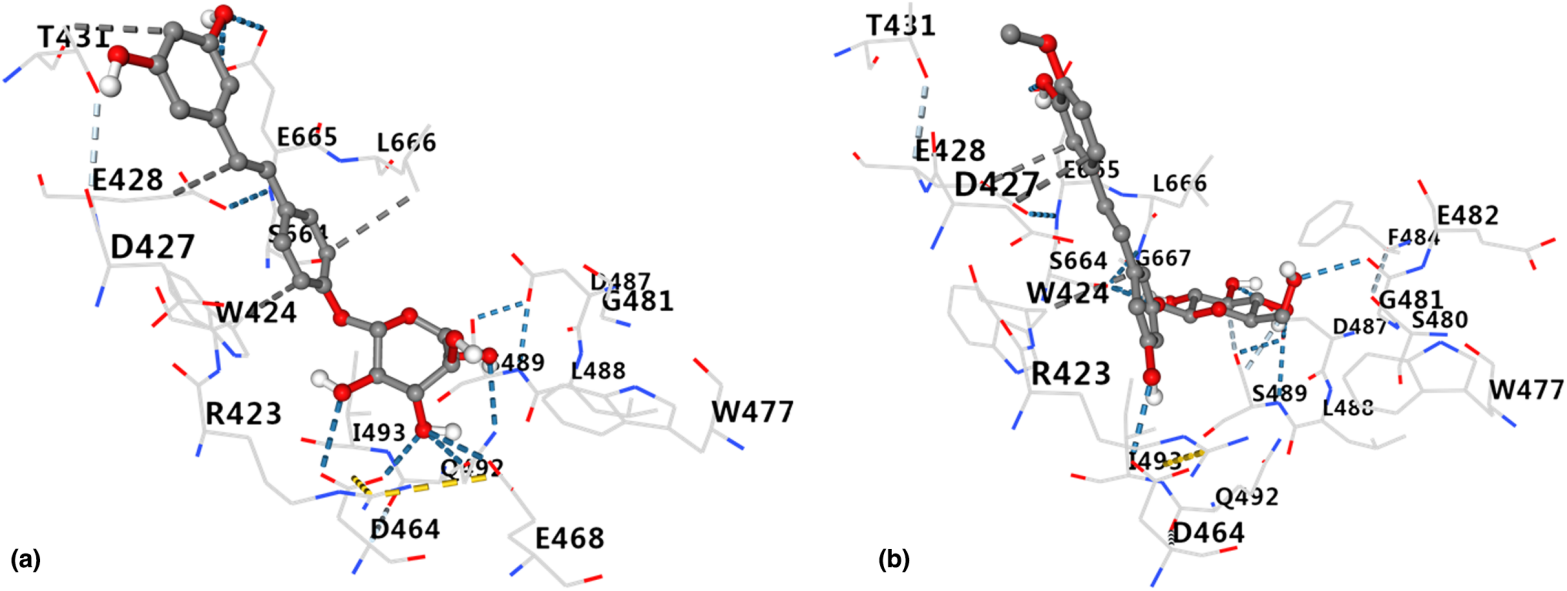
Binding of Resveratroloside (a) and rhaponticin (b) to amino acid residues of trimethylamine (TMA)‐lyase.

All stilbenes can bind to TMA‐lyase, and Resveratroloside and rhaponticin were the top two compounds with the highest Vina score (Table 1 and Figure 2). Figure 3 depicts the binding of Resveratroloside and rhaponticin to the TMA‐lyase amino acid residues. Resveratroloside had the highest Vina score, indicating that it is the most active chemical and may be utilized as a lead compound for structural modification.

**TABLE 1:** Docking of stilbene aglycones with trimethylamine (TMA)‐lyase

Chemicals	Vina score	Cavity score	Center (*x*, *y*, *z*)	Size (*x*, *y*, *z*)
Resveratroloside	−7.2	1074	−24, 19, −73	26, 26, 26
Rhaponticin	−7.1	1074	−24, 19, −73	26, 26, 26
2,3,5,4′‐Tetrahydroxystilbene 2‐O‐glucoside	−7.0	1074	−24, 19, −73	22, 22, 22
Polydatin	−7.0	1074	−24, 19, −73	25, 25, 25
Oxyresveratrol	−6.5	1074	−24, 19, −73	21, 21, 21
3,4,5,4′‐Tetramethoxystilbene	−6.4	1074	−24, 19, −73	23, 23, 23
Resveratrol	−6.3	1074	−24, 19, −73	21, 21, 21
Piceatannol	−6.3	1074	−24, 19, −73	22, 22, 22
Pinosylvin	−6.3	1074	−24, 19, −73	21, 21, 21
Rhapontigenin	−6.3	1074	−24, 19, −73	22, 22, 22
4‐Hydroxystilbene	−6.2	1074	−24, 19, −73	21, 21, 21
Pterostilbene	−6.1	1074	−24, 19, −73	22, 22, 22

## 4 CONCLUSIONS

These stilbene aglycones and glycosides can obviously affect the role of gut microbiota. Resveratrol can regulate the composition of gut microbiota, change the relative ratio of*Bacteroidetes*/*Firmicutes*, and reverse the imbalance of gut microbiota caused by high‐fat diet, reduce the elevated TMAO, and affect cardiovascular risk factors (Bird et al., 2017). Polydatin can improve the contents of valerate and caproic acid in feces by changing gut microbiota, further activate AMPK, improve hypercholesterolemia, and improve lipid metabolism (Zhao et al., 2022). Piceatannol can significantly change the gut microbiota of HFD‐induced obesity animal model, and significantly increase *Firmicutes* and *Lactobacillus* and decrease *Bacteroidetes* (Tung et al., 2016). Pterostilbene can significantly change the gut microbiota of Zucker (fa/fa) rats, significantly decrease the levels of*Firmicutes*, and increase Verrucomicrobia phyla (Etxeberria et al., 2017).

On the basis of affecting the gut microbiota, the results combined with molecular docking can significantly affect TMA‐lyase. Stilbenes have beneficial effects on the prevention and treatment of CHD, as well as the management of gut microbes, and are therefore prospective preventive medicines for CHD via TMA‐lyase inhibition. CB‐Dock was used to determine how well stilbenes inhibit TMA‐lyase. Resveratroloside and rhaponticin had a high affinity for TMA‐lyase, indicating that they were the most active and might be employed as lead compounds for structural alteration. In this review, the possible action process of TMA‐Lyase lead compound was described, which further affects the occurrence and development of CHD, providing an important theoretical basis for the discovery of innovative novel drugs for CHD. In the future, we will carry out in vivo and in vitro experimental research on the basis of this review to clarify its protection of CHD by reducing TMA‐lyase activity.

## ACKNOWLEDGMENT

This work was supported by the Anhui Province Education Department Nature and Science Fund. Key Project of China, Hefei, China (No. KJ2018ZD065) and the Special Fund for Modern Chinese medicine technology innovation and social service team of Bozhou Vocational and Technical College (Nos. yptd001 and ypzk002).

## CONFLICT OF INTEREST

The authors declare that they have no conflict of interest.

## Data Availability

The data used to support the findings of this study are available from the corresponding author upon request.
